# Association mapping of resistance to rice blast in upland field conditions

**DOI:** 10.1186/s12284-016-0131-4

**Published:** 2016-11-09

**Authors:** Louis-Marie Raboin, Elsa Ballini, Didier Tharreau, Alain Ramanantsoanirina, Julien Frouin, Brigitte Courtois, Nourollah Ahmadi

**Affiliations:** 1 CIRAD, UPR AIDA, TA B-115/02, Avenue Agropolis, 34398 Montpellier Cedex 5, France; 2 Montpellier SupAgro, UMR BGPI, TA A-54/K, Campus international de Baillarguet, 34398 Montpellier Cedex 5, France; 3 CIRAD, UMR BGPI, TA A-54/K, Campus international de Baillarguet, 34398 Montpellier Cedex 5, France; 4 FOFIFA, BP 230, Antsirabe, 110 Madagascar; 5 CIRAD, UMR AGAP, TA A-108/03, Avenue Agropolis, 34398 Montpellier Cedex 5, France

**Keywords:** Rice, Blast disease, Genome-Wide Association Study, Quantitative resistance, Qualitative resistance

## Abstract

**Background:**

Rice blast is one of the most damaging disease of rice. The use of resistant cultivars is the only practical way to control the disease in developing countries where most farmers cannot afford fungicides. However resistance often breaks down. Genome wide association studies (GWAS) allow high resolution exploration of rice genetic diversity for quantitative and qualitative resistance alleles that can be combined in breeding programs to achieve durability. We undertook a GWAS of resistance to rice blast using a tropical japonica panel of 150 accessions genotyped with 10,937 markers and an indica panel of 190 accessions genotyped with 14,187 markers.

**Results:**

The contrasted distribution of blast disease scores between the indica and tropical japonica groups observed in the field suggest a higher level of quantitative resistance in the japonica panel than in the indica panel. In the japonica panel, two different loci significantly associated with blast resistance were identified in two experimental sites. The first, detected by seven SNP markers located on chromosome 1, colocalized with a cluster of four NBS-LRR including the two cloned resistance genes *Pi37* and *Pish*/*Pi35*. The second is located on chromosome 12 and is associated with partial resistance to blast. In the indica panel, we identified only one locus associated with blast resistance. The three markers significantly detected at this locus were located on chromosome 8 in the 240 kb region carrying *Pi33*, which encompasses a cluster of three nucleotide binding site-leucine-rich repeat (NBS-LRRs) and six LRR-kinases in the Nipponbare sequence. Within this region, there is an insertion in the IR64 sequence compared to the Nipponbare sequence which also contains resistance gene analogs. *Pi*33 may belong to this insertion. The analysis of haplotype diversity in the target region revealed two distinct haplotypes, both associated with *Pi33* resistance.

**Conclusions:**

It was possible to identify three chromosomal regions associated with resistance in the field through GWAS in this study. Future research should concentrate on specific indica markers targeting the identified insertion in the *Pi33* zone. Specific experimental designs should also be implemented to dissect quantitative resistance among tropical japonica varieties.

**Electronic supplementary material:**

The online version of this article (doi:10.1186/s12284-016-0131-4) contains supplementary material, which is available to authorized users.

## Background

Blast is one of the most damaging diseases of rice (*Oryza sativa*), which is the staple crop for more than half the world population. Rice blast disease is distributed in all rice growing countries and can cause severe yield loss up to 100 % (Kato 2001). Each year blast destroys harvests that could feed 60 million people, at a cost of some $66 billion (Pennisi 2010). Blast is caused by the fungal pathogen *Magnaporthe oryzae*, ranked the most important fungal pathogen in molecular plant pathology (Dean et al. 2012). So far, around 100 blast resistance genes involved in qualitative, race specific resistance have been identified in rice germplasm (Sharma et al. 2012) and 21 of them have been cloned (Liu et al. 2014). The resistance gene *Pi33* is a particular focus of interest for our research team. The *Pi33*/*Ace1* interaction is an exception to the classic resistance gene/avirulence gene model. Unlike most known avirulence genes that directly encode small proteins, the avirulence gene *Ace1* encodes a cytoplasmic biosynthetic protein responsible for the production of a secondary metabolite recognized specifically by rice plants bearing *Pi33* (Collemare et al. 2008). *Pi33* was fine mapped (Berruyer et al. 2003; Ballini et al. 2007) but still remains to be cloned. Hundreds of QTLs involved in quantitative blast resistance have also been detected (Ballini et al. 2008) but with the exception of *Pi21* (Fukuoka et al. 2009), very few have been fully characterized. Blast fungicides are often too expensive for smallholder farmers like those in Madagascar, and using resistant cultivars is the only practical way of controlling the disease. However, resistance breakdown following adaptation of the fungus has frequently been observed (Ou 1985) and breeding durably resistant rice cultivars remains a challenge. Molecular breeding approaches facilitate the identification and deployment of resistance genes (Ashkani et al. 2015) and effective and durable resistance would be best achieved by combining both quantitative and qualitative resistance (Ballini et al. 2008; Boyd et al. 2013).

Rapid progress has recently been made in understanding plant pathogen interactions and disease resistance (reviewed by Jones and Dangl 2006; Dangl et al. 2013 and by Boyd et al. 2013). The molecular interactions specifically involved in the rice-blast pathosystem were reviewed by Liu et al. (2014). Broadly speaking, plants have developed two strategies to counter attacks by pathogens. The first line of active plant defense involves the recognition of pathogen-associated molecular patterns (PAMPs). PAMPs are essential and highly conserved pathogen structures such as the flagella of bacteria or the chitin found in fungi cell walls. They trigger general plant defense responses, referred to as PAMP-triggered immunity (PTI). Pattern recognition receptors (PRR) are receptor kinases or receptor-like proteins located on the cell surface that recognize these conserved pathogen signatures (Zipfel 2014). However, plant pathogens have developed the ability to secrete effector proteins into plant cells that suppress PTI through the inhibition of signaling pathways or the synthesis of defense compounds by the host plant (Alfano 2009). In response, plants implement a second line of defense involving the recognition of some of these effector proteins, called avirulence proteins, by nucleotide binding and leucine-rich repeat proteins (NB-LRR) encoded by resistance genes. This effector-triggered immunity (ETI), governed by a gene-for-gene interaction, induces plant defense responses that are regulated by a complex network of signaling pathways mainly involving three signaling molecules: salicylic acid, jasmonic acid and ethylene (Kunkel and Brooks 2002).

GWASs rely on linkage disequilibrium to detect associations between DNA sequence variations (markers) and phenotypes. The usually broad diversity of variety panels used for GWAS (germplasm bank collections, breeding materials created and evaluated during crop improvement or cultivar registration programs) gives access to a greater number of alleles (Yu et al. 2006). Likewise, having accumulated many more recombination events than bi-parental mapping populations, such panels ensure finer genetic resolution. GWAS should be particularly efficient for inbred lines, such as in rice, because after lines have been densely genotyped or completely sequenced, it becomes possible to analyze an unlimited number of traits in genetically identical material across a wide range of environments (Nordborg and Weigel, 2008). However, false-positive associations between markers and phenotypes may arise in GWAS because of population structure and cryptic relatedness. Population admixture creates Linkage Disequilibrium between unlinked loci and needs to be controlled in statistical analysis (Yu et al. 2006). In addition, GWAS may not be able to detect the phenotypic effects of rare alleles (present at very low frequencies in the populations studied) and GWAS detection power may decrease in the case of loci involving multiple allelic variants (Morris and Kaplan 2002), which may explain why GWAS have only unraveled a small portion of phenotypic variance (Zuk et al. 2014). Despite these constraints, GWAS have been successfully used to dissect complex traits in many crop species including maize (Buckler et al. 2009), sorghum (Morris et al. 2013) and rice (Huang et al. 2010; Zhao et al. 2011; Huang et al. 2012; Courtois et al. 2013; Huang et al. 2015; Si et al. 2016; Yano et al. 2016). Incorporation of GWAS information in genomic selection models has also proved able to improve the accuracy of predictions and should consequently be used in rice breeding programs (Spindel et al. 2015; Spindel et al. 2016).

The availability of high quality reference genome sequences for rice, along with recent advances in our understanding of the molecular events occurring in plant-pathogen interactions in combination with high throughput GWAS can be a very powerful tool for the identification of candidate alleles involved in natural variation in disease resistance. GWAS have already been used to assess the genetic variability underlying resistance of plants to various pathogens (Boyd et al. 2013) including spring wheat resistance to stripe rust (Maccaferri et al. 2015), maize quantitative resistance to northern leaf blight (Poland et al. 2011) or southern leaf blight (Kump et al. 2011). Rice resistance to blast has been investigated through GWAS in a limited number of studies. Wang et al. (2014) used 366 indica varieties inoculated with 16 different *M. oryzae* isolates to dissect blast resistance using a high resolution GWAS approach. These authors identified 30 loci associated with resistance. Kang et al. (2016) identified 97 loci associated with blast resistance using a representation of the five major subpopulations of *O. sativa (RDP1 panel)* and spray inoculation with five diverse *M. oryzae* isolates. Rice resistance to blast, among other traits, was also investigated through GWAS by Zhao et al. (2011) using 413 rice accessions artificially inoculated with a mixture of three *M. oryzae* isolates. These authors identified nine loci associated with resistance in at least one of four rice subpopulations. Huang et al. (2015) also used GWAS to dissect blast resistance among 38 other agronomic traits in a panel of 1495 hybrid rice varieties and identified four loci associated with resistance to blast. Sixteen loci associated with field resistance to blast in irrigated conditions were identified through GWAS by Zhu et al. (2016) using the RDP1 diversity panel. Here, we report the results of a genome-wide association study of blast resistance evaluated in naturally occurring blast epidemics at two upland rice breeding sites that are hot spots for blast. The rice germplasm scored for blast resistance was composed of a tropical japonica panel of 150 accessions genotyped with 10,937 markers and an indica panel of 190 accessions genotyped with 14,187 markers.

## Results

### Field evaluation of blast resistance

**Fig. 1:**
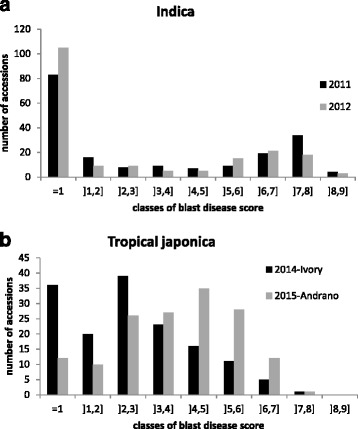
Distribution of blast disease scores measured **a**) in the indica panel in 2011 and 2012 in Andranomanelatra and **b**) in the japonica panel in 2014 in Ivory and 2015 in Andranomanelatra

The distributions of blast disease scores for the indica panel in 2011 and 2012 at the same location, a breeding station located at an altitude of 1635 m asl, are presented in Fig. 1a. The distributions were similar in the two study years. Based on a 1–9 scoring system on leaves (from 1 absence of symptoms up to 9 corresponding to lesion area >85 %), we considered all accessions with a score below or equal to two (corresponding to incompatible reactions) as resistant, and the remainder as susceptible. Twenty-one accessions out of 190 changed status from resistant to susceptible (highly susceptible with scores >5 in 8 cases) or the reverse in the two scoring years (Additional file 1: Table S1). The distributions were clearly bimodal with a contrasted separation between resistant accessions and susceptible accessions. When not resistant, most of the accessions appeared to be highly susceptible with scores >5. The distributions of blast disease scores measured in the japonica panel in 2014 at the mid-altitude breeding station (900 m asl) and in 2015 at the high altitude breeding station (1635 m asl) are presented in Fig. 1b and in Additional file 2: Table S2. The distributions differed in the two conditions. In 2014, the distribution was slightly bimodal with perceptible segregation between resistant accessions (scores ≤2) and susceptible accessions, whereas in 2015, the distribution was almost unimodal and normally distributed. According to the two scores, the epidemiological conditions appeared to differ in terms of pathogen populations, as most accessions scored as resistant at the Ivory experimental site in 2014 were not scored as resistant at the Andranomanelatra experimental site in 2015. However, the blast disease scores measured in 2011 and 2015 on 18 differential lines (i.e. carrying different resistance genes) both in Andranomanelatra were very similar. The resistance genes to blast *Pi2*, *Pi9*, *Pi33*, *Pita* ^2^, *Pizt* appeared to be efficient whereas *Pi1*, *Pia*,*Piz*, *Pii*, *Piks*, *Pikh*, *Pikp*, *Pit*, *Pita* and *Pish* appeared to be overcome (Additional file 3 : Table S3). These results suggest pathogen populations vary more with location than with year. Broad sense heritability of blast resistance scores was very high (h^2^ = 0.93 with only two repetitions) as measured in the 2015 experimental design. Thus our scoring of blast disease resistance should be accurate even in absence of replications.

### Extent of LD and genome coverage

On average across all chromosomes, LD reached half of its original value at around 90 Kb for the indica panel and around 200 Kb for the tropical japonica panel (Additional file 4: Figure S1). LD dropped to an r^2^ value of 0.2 within 140 kb in the indica panel and 440 kb in the tropical japonica panel. LD was rather high in both panels. LD extended over longer distances (almost three times longer) in the tropical japonica panel than in the indica panel. These differences between subspecies were also reported by Mather et al. (2007). As a consequence, a much larger number of markers would be required to achieve the same genome coverage for the indica panel than for the japonica panel. In this study we used 14,187 markers for the indica panel and 12,438 markers for the japonica panel. Marker density was heterogeneous along chromosomes particularly in the japonica panel. In the indica panel, we counted 16 gaps of over 0.5 Mb between markers, and one of them on chromosome 2 reached 3.5 Mb. In the japonica panel, we counted 61 such gaps, and one of them on chromosome 5 reached 2.7 Mb. Nevertheless, the average distance between adjacent markers of 24 kb in the indica panel and of 30 kb in the tropical japonica panel was below LD decay in both cases and the average genome coverage was considered fair enough to detect and map resistance loci.

### Genome-Wide Association Study of rice blast resistance

**Fig. 2:**
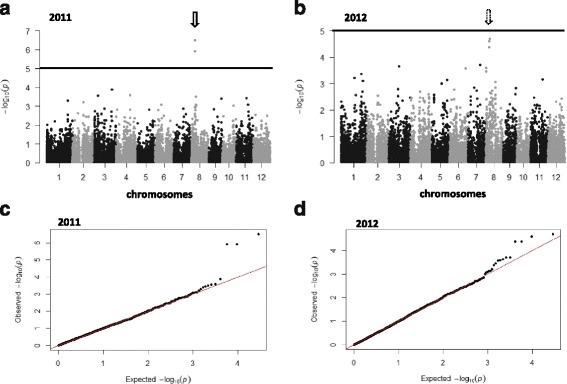
Manhattan plots of the markers associated with rice blast disease resistance in the indica panel in Andranomanelatra in **a**) 2011 and **b**) 2012. X axis show markers along the 12 rice chromosomes and Y axis shows the negative log10-transformed *p*-values for each association. Q-Q plots of the markers associated with rice blast disease resistance in the indica panel in Andranomanelatra in **c**) 2011 and **d**) 2012. *Full line*: *P* = 1.10^−5^

**Fig. 3:**
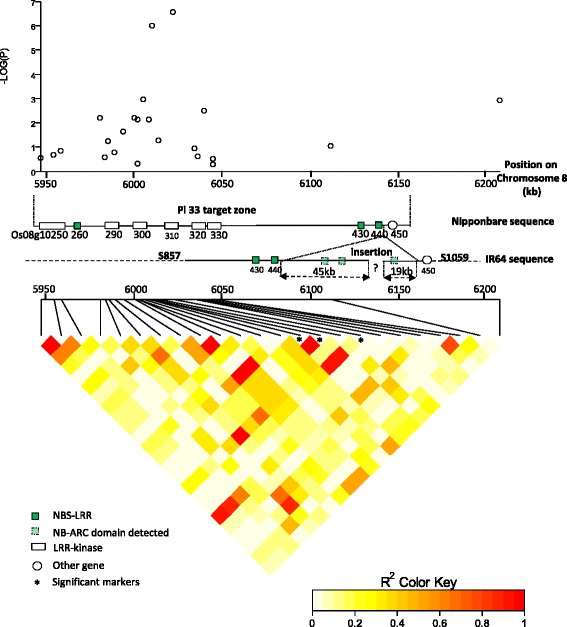
Manhattan plot and pattern of linkage disequilibrium (LDheatmap) in the *PI33* target zone. The gene organization of this zone in Nipponbare reference sequence and IR64 reference sequence is also presented. The represented sequence segment is 250 kb long in Nipponbare

In the indica panel, in 2011, we identified only one locus significantly associated with blast resistance (Fig. 2a) using a q-value of 0.1 as the genome-wide significance threshold. This locus involved three markers located on the short arm of chromosome 8 at positions Chr8_6008697 (*P* = 1.2 × 10^−6^ and q-value = 5.6 × 10^−3^), Chr8_6008700 (*P* = 1.2 × 10^−6^ and q-value =5.6 × 10^−3^) and Chr8_6020507 (*P* = 3.17 × 10^−7^ and q-value = 4.5 × 10^−3^). The Q-Q plot showed very little deviation from the identity line (Fig. 2c) except for the few markers with low *p* values, suggesting that our conditions are good enough to detect true associations. In 2012, the same set of markers showed the highest probability (q-value = 0.1478), although slightly above the threshold retained for significance (Fig. 2b). The Q-Q plot also showed little deviation from the identity line (Fig. 2d). These three markers are positioned within the region carrying *PI33* previously identified by genetic mapping by Ballini et al. (2007), (Fig. 3). This region of 240 kb (0.4 cM) delimited by microsatellite markers RMSPi33–53 and RM3507 encompasses two clusters of resistance gene analogs in the reference genome of Nipponbare (which does not carry *Pi33*). The first cluster is composed of six kinases and one NB-LRR (between Os08g10250 and Os08g10330, Fig. 3). The second cluster is composed mainly of NB-LRR (between Os08g10430 and Os08g10450). In the second cluster, an insertion was identified in the IR64 (which carries *PI33)* sequence compared to the Nipponbare sequence that may exceed 64 kb and contains signaling motifs characteristic of NB-LRRs (Fig. 3). This insertion corresponds to at least 45 kb at the end of scaffold_857 (3900 kb), and to 19 kb at the beginning of scaffold_1059 (178 kb) in IR64 (Schatz et al. 2014; Schatz personal communication, Fig. 3). Recalling SNPs after alignment to IR64 scaffolds identified a total of 65 markers in the target zone, two of which belonged to the insertion zone. However, no new markers associated with blast resistance were detected.

**Fig. 4:**
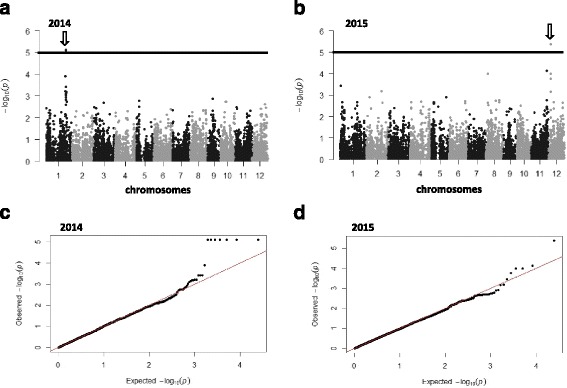
Manhattan plots of the markers associated with rice blast disease resistance in the japonica panel in **a**) 2014 in Ivory and **b**) 2015 in Andranomanelatra. The X axis shows markers along the 12 rice chromosomes and the Y axis shows the negative log10-transformed *p*-values for each association. Q-Q plots of the markers associated with rice blast disease resistance in the japonica panel in **c**) 2014 in Ivory and **d**) 2015 in Andranomanelatra. *Full line*: *P* = 1.10^−5^

**Fig. 5:**
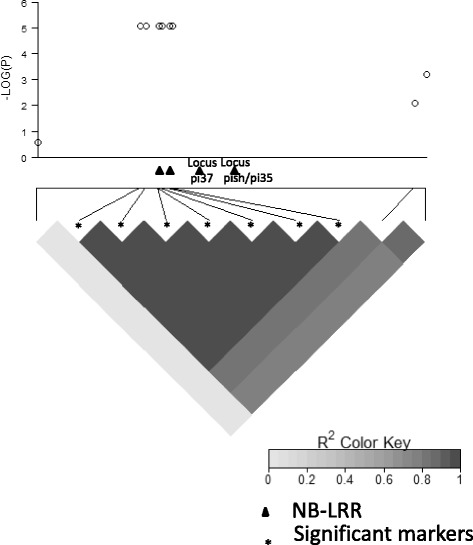
Manhattan plot and pattern of linkage disequilibrium (LDheatmap) around the Pi35, PI37, Pish cluster of NB-LRR. The sequence segment shown is 295 kb long

In the japonica panel, we identified two distinct loci significantly associated with blast resistance using a q-value of 0.1 as the genome-wide significance threshold. In 2014, one locus was detected in the middle altitude breeding station and the other one was detected in 2015 in the high altitude breeding station (Fig. 4). The locus detected in 2014 conditions involved seven markers in full LD on the long arm of chromosome 1 between positions 33076887 and 33101162 (*P* = 7.89 × 10^−6^ and q-value = 1.4 × 10^−2^ for all seven markers). These seven markers delimit a segment overlapping two NBS-LRR genes (Os01g57270 and Os01g57280; Fig. 5). They are adjacent to two other NBS-LRR (Os01g57310 and Os01g57340). The first one is allelic to *Pi37* (Lin et al. 2007) and the other one corresponds to the position of both *Pish* and *Pi35*. Os01g57340 corresponds to the sequence of *Pish* in Nipponbare (Takahashi et al. 2010). *Pi35* is allelic to Pish and confers quantitative resistance to blast even though it belongs to the NBS-LRR class (Fukuoka et al. 2014). The locus detected in 2015 conditions involved only one marker on chromosome 12 at position 4423506. The closest defense gene candidate to this marker would be the Thioredoxin *Ostrxm* (Os12g08730) located at position Chr12_ 4463096 (at a distance of 39 kb from the detected marker). This gene has also been reported to be essential for chloroplast development and rice growth (Chi et al. 2008) and thioredoxin proteins play important roles in defense against pathogens (Li et al. 2016).

### Analysis of haplotype diversity at each detected locus

We used a set of closely linked markers to analyze the relationship between haplotype diversity and phenotypes for blast resistance at the loci identified through GWAS. In the indica panel, six markers surrounding the most significant marker Chr8_6020507 revealed seven distinct haplotypes among the 190 varieties (Table 1). A Kruskall-Wallis test showed that the blast disease resistance scores of the populations of varieties corresponding to each haplotype were different both in 2011 and 2012. Two distinct haplotypes were associated with blast resistance: the TTAAAG haplotype grouping 39 varieties and the AATCAA haplotype grouping 27 varieties (Additional file 1: Table S1). The variety Taichung Native 1, which was previously identified as carrying *Pi*33 (Ballini et al. 2007), belongs to the TTAAAG haplotype group, while the variety IR64, which was used for *Pi*33 fine mapping (Ballini et al. 2007), belongs to the AATCAA haplotype group. The same structure was observed among 2809 accessions in the 3000 rice genomes project (Li et al. 2014; Additional file 5: Figure S2) using 1130 SNP markers within the target region for *Pi*33. Two distinct haplotype groups were identified that may both be associated with *Pi*33 resistance (Additional file 6: Table S4). The first group comprised 253 accessions including Taichung native 1 and Tsai Yuan Chon, also identified as carrying *Pi*33 (Ballini et al. 2007). A total of 97 % of the accessions in this group of haplotype were identified as indica and they represent 14 % of the total number of indica accessions. The second group comprised 70 accessions including IR64 and IR5657-33-2, which have also been identified as carrying *PI33* (Ballini et al. 2007). A total of 99 % of the accessions in this group of haplotypes were identified as indica and they represent 4 % of the total number of indica accessions.

**Table 1:**
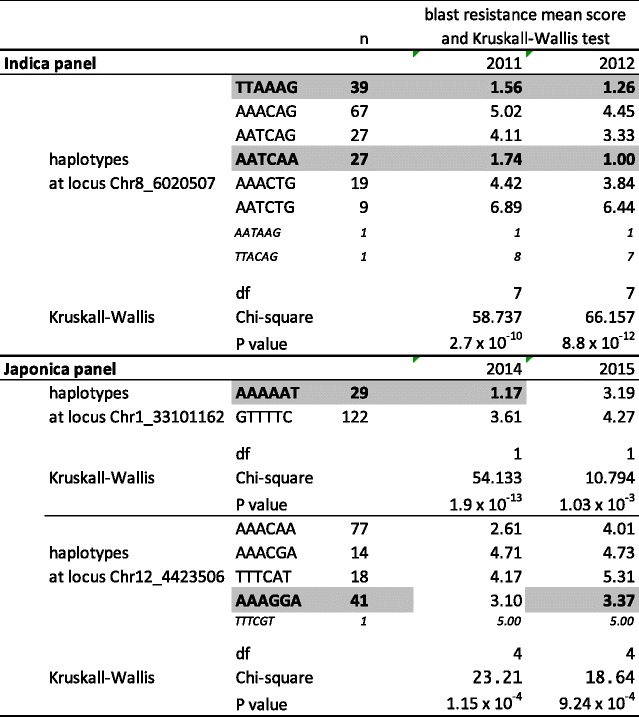
Mean blast resistance score per haplotype class at each candidate locus. Kruskall and Wallis test

Haplotypes putatively associated with resistance are highlighted in grey

In the japonica panel, at the locus detected on chromosome 1 in 2014, in the middle altitude breeding station, we used six out of the seven significant markers to construct haplotypes. These six markers revealed only two haplotypes (Table 1). We did not use any additional markers adjacent to the set of detected markers to analyze haplotype diversity because the next marker was positioned at a distance of 200 Kb away, a distance encompassing over 15 putative genes. A Kruskall-Wallis test showed that the blast disease resistance scores of the populations of varieties corresponding to each of these haplotypes were highly significantly different in 2014 and were also slightly different in 2015 conditions. In 2015, the difference was quantitative in nature whereas in 2014 conditions, it was qualitative (i.e. resistance versus susceptibility). The haplotype associated with resistance comprised 29 varieties.

In the japonica panel, at the locus detected on chromosome 12 in 2015 conditions, six markers surrounding the most significant marker Chr12_4423506 revealed five distinct haplotypes among the 151 varieties (Table 1). In this, case the variation between haplotype classes was quantitative rather than qualitative and the Kruskall-Wallis test showed that the blast disease resistance score of the populations of varieties corresponding to each of these haplotypes differed significantly.

The AAAGGA haplotype associated with a putative QTL for resistance comprised 41 varieties. In 2014 conditions, although the Chr12_4423506 marker was not significant according to the GWAS, the Kruskall-Wallis test indicated a significant difference in blast disease resistance scores between haplotype classes. However, in this case, the AAACAA haplotype was the most resistant mainly because this haplotype class contains nearly all the resistant varieties associated with the resistant haplotype identified on chromosome 1.

## Discussion

### Tropical japonica varieties harbor a higher level of partial resistance than indica varieties

The distribution of blast disease scores was contrasted between the indica and tropical japonica groups. In the indica panel, a bimodal distribution was observed with two clearly opposite peaks, one corresponding to resistant varieties (score 1 or 2) and one corresponding to highly susceptible varieties (score >5). This distribution points to the segregation of major resistance genes as well as to the near absence of quantitative resistance in the indica panel (Fig. 1a). On the other hand, in the tropical japonica panel disease scores were distributed in a more or less Gaussian shape around intermediate disease scores (Fig. 1b). This shows there is a higher level of quantitative resistance within the japonica panel than within the indica panel and corroborates the existence of a difference in preformed defense expression between indica, temperate japonica and tropical japonica subgroups (Vergne et al. 2010). Partial resistance along with constitutive (i.e. before infection) expression of defense related genes was highest in the tropical japonica group. Most of the tropical japonica varieties also presented a distinct pattern of expression of constitutive defense related genes compared to the other varieties, suggesting that this group may possess a particular preformed defense system (Vergne et al. 2010).

### Limitations of detection power and resolution of GWAS in our conditions

We identified three loci associated with resistance in total and one locus per panel x environment combination. This is far less than the 97 loci in total identified by Kang et al. (2015) using five blast strains. The great majority (75/97) of the loci associated with blast resistance detected by these authors were specific to one strain. The number of loci we were able to identify was also less than the 30 identified by Wang et al. (2014) in an indica panel using 16 blast strains. More in line with our results, Zhao et al. (2011) only detected a total of nine loci (five in their indica panel, none in their tropical japonica panel) using a mixture of three blast strains and Zhu et al. (2016) detected 16 loci for resistance to blast in field conditions over three different environments in the RDP1 panel. Under our natural infestation conditions, the genetic structure of *M. oryzae* populations was complex, with a broad virulence spectrum as observed in our panel of differential lines. Such conditions drastically reduced the number of R genes that could potentially be detected. However, particularly in our tropical japonica panel, the near absence of complete resistance observed should have provided the right situation to detect quantitative resistance. Unfortunately, only one QTL was detected. The size of the tropical japonica panel (151 varieties) may have been too small to detect the usually small effects of QTLs. Genome wide investigations that specifically target quantitative resistance should be further considered in the future, preferentially in the tropical japonica background with the objective to better understand the genetic architecture of quantitative resistance to blast. Although the accumulation of quantitative resistance loci could contribute to achieve durable resistance (Poland et al. 2008), its molecular basis is still largely unknown in contrast to the gene-for-gene resistance model. To perform GWAS, we used 14,187 markers for the indica panel and 12,438 for the japonica panel. Although recently, some GWAS involved over a million SNP markers (Huang et al. 2015), given the extent of linkage disequilibrium in both panels, the genome coverage should have been sufficient to detect most of the loci involved in rice blast resistance. The drawback is that the resolution power of GWAS should be limited as many candidate genes may be detected in a same LD block (Yano et al. 2016). Still, a higher density of markers may allow localizing with more precision causative genes and polymorphisms. For example, Kang et al. (2015) clearly improved their mapping resolution when they compared a 44 K SNP dataset and a 700 K SNP dataset for the analysis of a same resistance locus reducing their target zone from 220 kb to 20 kb. To increase our detection power, we also need to work with a larger number of accessions, which would enable us to capture more low-frequency resistant alleles (Zuk et al. 2014). Multiple alleles may exist at single resistance loci within populations with a broad genetic base, as we observed at the *Pi33* locus in our indica panel. It may therefore be useful to complement GWAS based on single biallelic markers with GWAS based on haplotypes (Lorenz et al. 2010). In the context of an autogamous plant species such as rice, this should be straightforward as it would not involve the complication of inferring the linkage phase of the markers.

### Candidate genes identified through GWAS

In the indica background, we identified a set of three markers associated with blast resistance and located within the 240 kb target region for the resistance gene to blast *Pi33* (Fig. 3). *Pi33* was fine mapped by Ballini et al. (2007) but remains to be cloned. In the Nipponbare sequence (MSU v7), this target region encompasses a cluster of resistance gene analogs including six kinases and three NBS-LRRs. Unfortunately, in our panel, the marker density along the target zone was uneven with a clear lack of markers in the second half of the target zone. The great majority of cloned resistance genes to rice blast belong to the NBS_LRR class (Liu et al. 2014). However, unlike most known avirulence genes that directly encode small proteins, the avirulence gene *Ace1* corresponding to *Pi33*, is responsible for the production of a secondary metabolite recognized by rice plants bearing *Pi33* (Collemare et al. 2008). Therefore we cannot rule out the possibility that *Pi33* is not a classical resistance gene. Our reasoning based on the Nipponbare reference sequence (MSU v7) may not be fully applicable in an indica background. Indeed, we identified an insertion in the IR64 sequence compared to the Nipponbare sequence that contains signaling motifs characteristics of plant resistance genes (Fig. 3). This configuration means the targeted gene might be localized within this insertion. However, recalling SNPs over the sequence of this insertion did not reveal new markers associated with blast resistance. Huang et al. (2015) also identified a locus associated with blast resistance in the vicinity of *Pi33* target zone. Their most significantly associated SNP marker was located at position Chr8_6161524, which is slightly outside the identified target zone but would nevertheless support the hypothesis that *PI33* belongs to the cluster of NB-LRR spanning between Os08g10430 and Os08g10450 (Fig. 3). The analysis of the haplotype diversity of the targeted *Pi33* zone revealed two clearly distinct haplotypes that may both be associated with *Pi33* resistance. Both haplotypes are specifically of indica origin and overall represent 18 % of the total number of indica accessions. This structure is also an indication that *Pi33* may belong to the indica-specific insertion.

In the japonica background, we identified a set of seven markers associated with blast resistance colocalized with a cluster of four NB-LRR including two cloned resistance genes *Pi37* and *Pish*/*Pi35*. However, it was impossible to pinpoint the causative polymorphism because of the low uneven marker density in that region in our panel (Fig. 5). In 2014, in the middle altitude experimental site, the resistance conferred by the resistant haplotype was complete. In the other environment (2015 in Andranomanelatra), where this resistance was defeated, the set of varieties carrying the resistant haplotype still presented a significantly higher level of quantitative resistance than the rest of the varieties. Partial resistance may in some cases be determined by the breakdown of the resistance conferred by R genes (Li et al. 1999; Fukuoka et al. 2014). Gallet et al. (2016) found a positive correlation between the narrow compatibility range of varieties and the reduced severity of successful infections, in line with the hypothesis that R genes may, in some instances, control both partial and complete resistance. We also identified a locus associated with partial resistance on chromosome 12. Chromosome 12 has been found to be rich in disease resistance genes (Rice Chromosomes 11 and 12 Sequencing Consortia 2005).

## Conclusions

GWAS was confirmed to be a very useful tool for mining resistance alleles in rice germplasm even though only three chromosomal regions associated with resistance in the field were identified in this study and although the organization of resistance genes in clusters made it difficult to unambiguously identify causative genes. This study revealed a complex picture at *Pi33* locus whith the existence of an indica specific insertion and the identification of two distinct haplotypes that may be associated with *Ace1* induced resistance. Further experiments should take advantage of the 3000 Rice Genomes high density genotyping to improve the resolution of GWAS. As tropical japonicas appear to be a potential source of interesting quantitative resistance alleles, this subgroup should be specifically explored through GWAS using larger panels and a higher marker density in a context of high blast disease pressure or using broad spectrum blast strains mixtures.

## Methods

### Plant material

The indica panel was composed of 190 accessions from 21 countries all over the world including 35 from Madagascar, 31 from Senegal, 27 from the Philippines, 19 from India and 12 from Mali for the most important groups. Ninety-six of these varieties are traditional varieties and 94 are improved varieties (Additional file 1: Table S1).

The tropical japonica panel was composed of 151 accessions from 28 countries all over the world including 23 from Philippines, 20 from Brazil, 18 from Indonesia, 17 from Madagascar and 15 from Ivory Coast for the most important groups (Additional file 2: Table S2).

Seeds of the accessions were obtained either from the *Centre de Ressources Biologiques Tropicales de Montpellier* or from the International Rice Research Institute (IRRI) gene bank. For each accession, the seeds were produced by single seed descent over two generations in a Cirad greenhouse in Montpellier to ensure that the samples were homogeneous.

### Disease assessment

The indica panel was evaluated for blast disease resistance in two distinct experiments in 2011 and 2012 in the same breeding station located close to the village of Andranomanelatra, 130 km south of the town of Antananarivo in Madagascar (19°47′ S, 47°06′ E) at an altitude of 1635 m a.s.l.

The japonica panel was evaluated for blast disease resistance in two distinct experiments in 2014 and 2015. In 2014, the experiment was conducted in a breeding station in the middle west of Madagascar (S19 33 16.8, E46 25 29.3) at an altitude of 900 m asl. In 2015, the experiment was located in Andranomanelatra like for the evaluation of the indica panel.

All the experiments were conducted under rainfed upland conditions. Blast infection occurred naturally but spreader rows of seven highly susceptible varieties (Rojofotsy, Manga vava, Molotra madame, Latsidahy, Latsibavy, FOFIFA 152 and FOFIFA 154) were used to ensure high, homogeneous disease pressure. Moreover, the blast disease pressure prevailing in Madagascar highlands and in our upland conditions is naturally high (Raboin et al. 2012). For each variety in both panels, two lines of 2.4 m were sown perpendicularly to the spreader rows. Blast symptoms were evaluated on leaves before the booting stage using a 1–9 scoring system (1, no symptoms; 2, few very small lesions; 3, higher number of small lesions some of which become elliptical; 4, expanding lesions with a total lesion area of up to 5 %; 5, lesion area 5–10 %; 5, lesion area 10–15 %; 6, lesion area 15–30 %; 7, lesion area 30–60 %; 8; lesion area 60–85 %; 9, lesion area >85 %) modified from the Standard Evaluation System for Rice (IRRI 1996).

These evaluations were performed without replication except in 2015 when we used a randomized block design with two replications to check the heritability of our blast resistance scoring. Broad-sense heritability (h^2^) was calculated from variance components obtained using the REML option of SAS PROC VARCOMP (SAS institute Inc.) where all factors were considered to be random (SAS, 2011) as: h^2^ = σ^2^_c_/(σ^2^_c_ + (σ^2^_e_/r)) where σ^2^_c_ and σ^2^_e_ are the variety and error variances, respectively, and r is the number of replications.

### Genotyping

Genomic DNA was extracted from the leaf tissues of a single plant from each accession using the MATAB method described in Risterucci et al. (2000) and then diluted to 100 ng/μl. Genotyping was conducted at Diversity Arrays Technology Pty Ltd. (DArT P/L), Australia, using a method of GBS combining Diversity Arrays Technology (DArT) and a next-generation sequencing technique called DArTseq™. The full methodology for marker production is described in Courtois et al. (2013). Missing data were estimated using Beagle v3.3, which enables the inference of haplotypes and imputation of sporadic missing data in large-scale genotype datasets (Courtois et al. 2013).

For the indica panel, we used 14,187 markers with no missing data and with a minimum frequency of minor allele of 4.2 % (8 out of 190). For the japonica panel we used 12,438 markers with a minimum frequency of minor allele of 5.3 % (8 out of 151).

### Linkage disequilibrium and marker trait association analysis

We used Tassel software to perform linkage disequilibrium and association analysis (Bradbury et al. 2007). These analyses were performed within each of the two sub-specific panels (i.e. indica and tropical japonica) separately. The R^2^ value of linkage disequilibrium was calculated for all possible marker pairs within each chromosome. We used the mixed linear model (MLM) with control of structure (Q) and kinship (K) to avoid spurious associations (Yu et al. 2006). The structure was taken into account through a principal component analysis (PCA) of the genotypic data (Price et al. 2006) computed with Tassel. We retained the first five principal components to build the Q matrix. The kinship matrix was also computed with Tassel. The mixed model analysis was conducted with the options of no compression and re-evaluation of the variances at each marker.

The critical values for assessing the significance of marker-trait associations were calculated using the q-value R package (Storey and Tibshirani 2003). The q-value package estimates the false discovery rate (FDR) from a collection of *p*-values. Only marker-trait associations with a q-value < 0.1 were considered significant. The threshold to declare a significant association was set to –log10 *P* = 5.

We ran the non-parametric Kruskal-Wallis test, based on ranks, to test if the different haplotype groups differed in their response to blast disease.

## Additional files

Additional file 1: Table S1. List of the 190 accessions included in the indica panel with their country of origin, their type (traditional or improved), their blast scores measured in 2011 and 2012 and, if need be, indication of the resistant haplotype they harbor at the *PI33* locus. (XLSX 16 kb)
Additional file 2: Table S2. List of the 151 accessions included in the japonica panel with their country of origin, their blast scores measured in 2014 in Ivory and 2015 in Andranomanelatra and, if need be, indication of the resistant haplotype they harbor at the Pi1 locus and the QTL locus at Chr12_4423506. (XLSX 26 kb)
Additional file 3: Table S3. Blast disease scores measured on 18 differential lines in Andranomanelatra in 2011 and 2015. (XLSX 9 kb)
Additional file 4: Figure S1. LD decay plot in the indica and in the japonica panels. (PPTX 147 kb)
Additional file 5: Figure S2. Analysis of haplotype diversity in the targeted *PI33* zone. A neighbor joining tree was constructed using 1130 SNP markers between positions Chr8_5998075 and Chr8_6214790 (with MAF > 2.5 % and less than 60 missing data per SNP) for 2809 accessions from the 3000 Rice Genomes Project (Li et al. 2014). (PPTX 68 kb)
Additional file 6: Table S4. List of the accessions from the 3000 Rice Genomes Project that belong to the two distinct haplotype groups (IR 64 group and Taichung Native 1 group) putatively associated with *PI33* resistance. (XLSX 127 kb)

## Abbreviations

DArT: Diversity arrays technology
ETI: Effector-triggered immunity
FDR: False discovery rate
GBS: Genotyping by sequencing
GWAS: Genome wide association study
LD: Linkage disequilibrium
MLM: Mixed linear model
NB-LRR: Nucleotide binding leucine rich repeat
PAMP: Pathogen-associated molecular patterns
PCA: Principal component analysis
PTI: PAMP-triggered immunity
QTL: Quantitative trait loci
R: Resistance
SNP: Single nucleotide polymorphism
