# Supplementary figures and images for: Association mapping of resistance to rice blast in upland field conditions
Source: Rice (N Y). 2016 Nov 9;9:59. doi: 10.1186/s12284-016-0131-4 (PMC5102990; doi:10.1186/s12284-016-0131-4)

## Slide 1
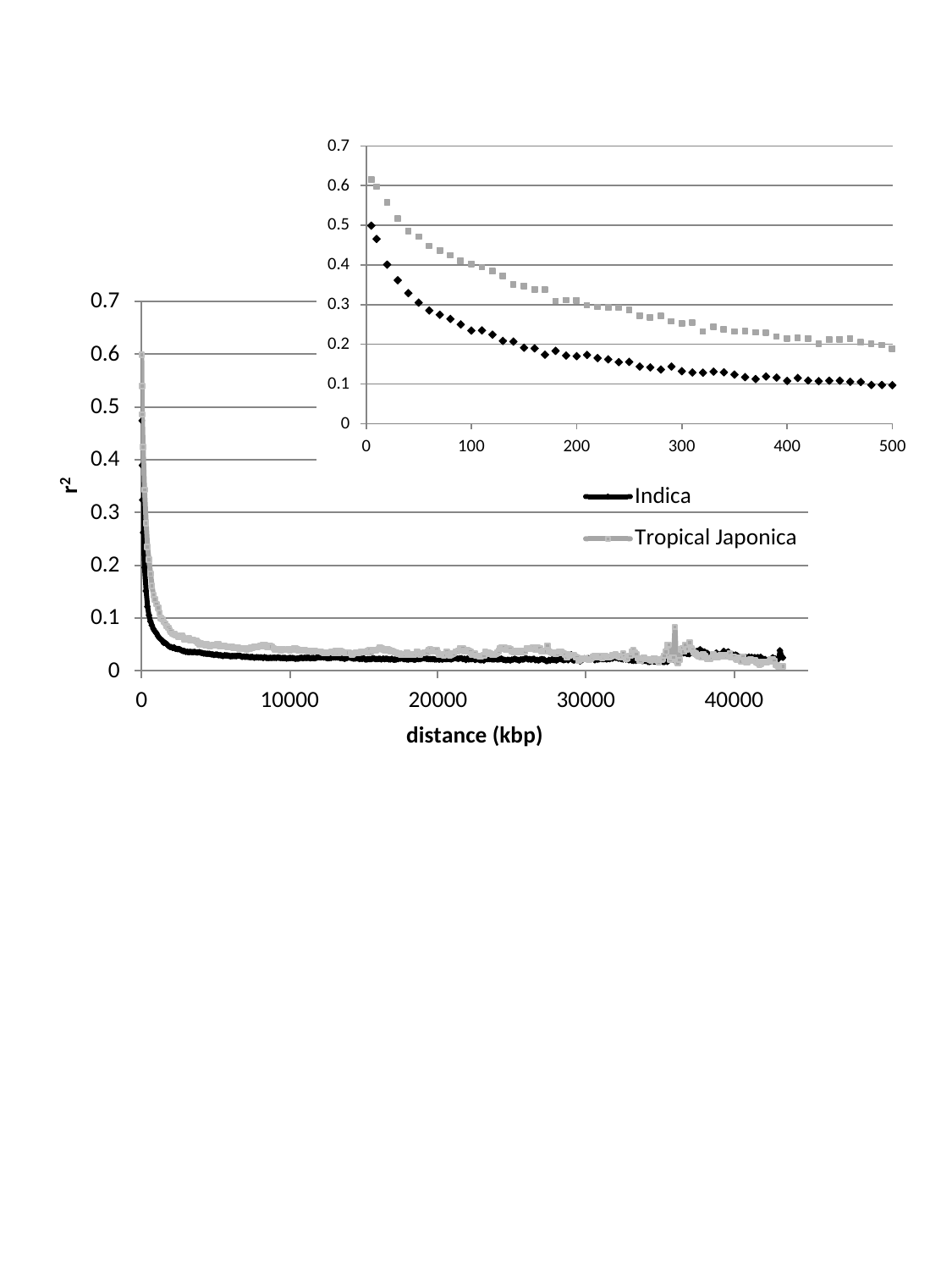

Supplement: Additional file 4: Figure S1. — LD decay plot in the indica and in the japonica panels. (PPTX 147 kb) [file 12284_2016_131_MOESM4_ESM.pptx]

## Slide 1
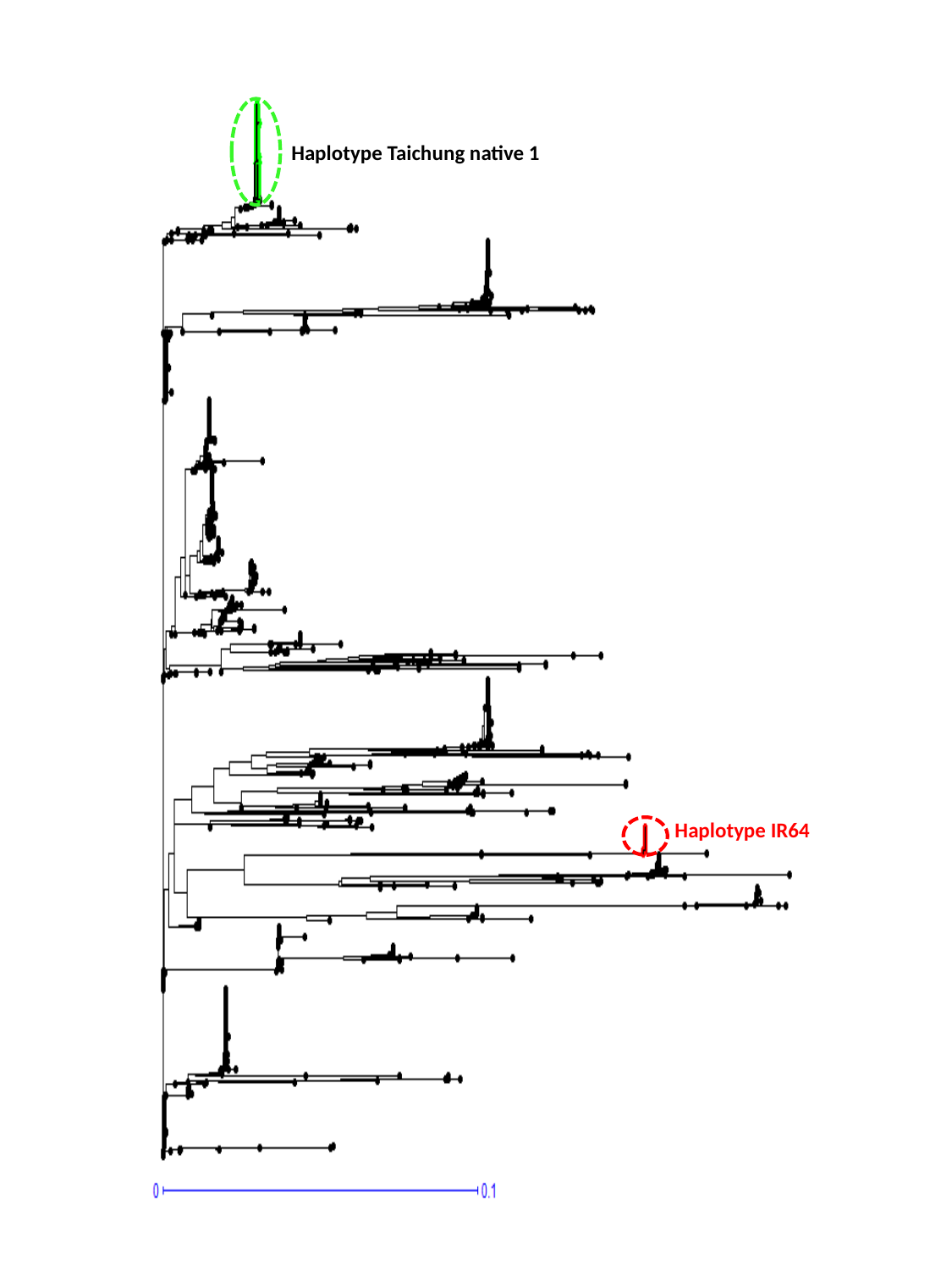

Haplotype Taichung native 1
Haplotype IR64

Supplement: Additional file 5: Figure S2. — Analysis of haplotype diversity in the targeted PI33 zone. A neighbor joining tree was constructed using 1130 SNP markers between positions Chr8_5998075 and Chr8_6214790 (with MAF > 2.5 % and less than 60 missing data per SNP) for 2809 accessions from the 3000 Rice Genomes Project (Li et al. 2014). (PPTX 68 kb) [file 12284_2016_131_MOESM5_ESM.pptx]
